# An effective balance is based on many pillars

**DOI:** 10.1093/icvts/ivac154

**Published:** 2022-06-01

**Authors:** Augusto D’Onofrio, Gino Gerosa

**Affiliations:** Division of Cardiac Surgery, University of Padova, Padova, Italy; Division of Cardiac Surgery, University of Padova, Padova, Italy

**Keywords:** Mitral valve repair, Micro-invasive cardiac surgery, Transapical neochords

We thank Dr Tomšič and colleagues from Leiden for their comments about our recently published study. Annular stabilization is the main concern of cardiac surgeons for long-term durability of transapical neochords implantation (NC) due to our well-established surgical technique. Transcatheter edge-to-edge mitral repair is always done with no annular stabilization but apparently, this is not seen as a major concern among interventional cardiologists who have demonstrated to be perseverant and keep on expanding indications and performing trials (REPAIR MR ClinicalTrials.gov Identifier: NCT04198870 and PRIMARY ClinicalTrials.gov Identifier: NCT05051033). As a matter of fact, so far data do not support the lack of mitral annulus stabilization as a potential cause of NC failure. In our entire experience with transapical-NC [1], failure has never been related to mitral annular enlargement. Furthermore, it has already been demonstrated that annular remodelling (reduction of annular diameter) occurs in patients undergoing this procedure [2]. Early referral and consequently early treatment of patients with degenerative mitral regurgitation (DMR) is likely going to reduce the need for annular stabilization. It is true that conventional surgery for DMR provides optimal results in terms of mortality and complications as well as of freedom from recurrent mitral regurgitation (MR) in centres of excellence that are dedicated and highly committed to this procedure [3] but the real world is a different thing [3, 4]. Our data show no statistical differences between conventional surgery and NC at follow-up in patients with type A anatomy in terms of recurrence of moderate MR (63.9% vs 74.6%), severe MR (79.3% vs 79%) and freedom from reoperation (79.7% vs 85%). This means that there is around a 10% difference of moderate MR recurrence at 5 years between NC and conventional surgery in well-selected patients. Now, the question is: is this 10% difference worth a micro-invasive operation with no cardiopulmonary bypass nor cardioplegic arrest and the possibility of a second operation (in case of failure) with no mediastinal adhesions? Given the (near) future perspective of percutaneous transseptal NC that has already been performed in humans [5] with several devices under development, do we really believe that this 10% difference (that includes learning curve and that will likely decrease in the future) will convince referring cardiologists, interventional cardiologists and our patients that open-heart surgery (although minimally invasive, robotic, video-thoracoscopic, etc.) is the preferred choice rather than a micro-invasive approach performed possibly through a femoral vein puncture? We must not forget what the history of transcatheter aortic valve implantation has taught us: a procedure with initial suboptimal results has become the gold standard in 10 years thanks to technology and commitment and surgeons have lost control over it. We advocate to keep this technology in our hands, to further develop these techniques, to critically and honestly evaluate results in order to offer our patients the entire range of different therapeutic options (conventional surgery, minimally invasive surgery and micro-invasive procedures) in a totally unbiased manner. An optimal choice should be based on an optimal balance of pros and cons of every therapeutic option together, of course, with patient’s will and expectations.
